# Editorial: Mass spectrometry to answer clinical questions: insights in oncology and health science research, vol II

**DOI:** 10.3389/fonc.2024.1419820

**Published:** 2024-06-11

**Authors:** Raphaela Menezes de Oliveira, Aline Maria Araújo Martins

**Affiliations:** ^1^School of Medicine, University of Brasilia, Brasilia, Brazil; ^2^The Scripps Research Institute, La Jolla, CA, United States

**Keywords:** mass spectrometry, oncology, health science, translational medicine, biomarker, biomedical research

## 1 Mass spectrometry: a powerful and versatile tool in the transition between research and healthcare

Initially consolidated in metabolomics and proteomics research studies, mass spectrometry (MS) is now on its way to becoming established as right-hand tool of modern clinicians. From prospection of potential biomarkers to tissue discrimination in surgery and tailor-made medicines, the technique has evolved to guide critical and immediate medical decisions with high sensitivity and specificity. Its broad scientific applicability favors the “bench-to-bedside” approach of Translational Medicine especially in the fields of Oncology, Health Sciences and Pharmaceuticals, leading the characterization of phenotypic molecular signatures even of single cells.

In this Research Topic, MS relevance in life research study was demonstrated by a series of four original research and one review article. Supporting MS natural potential as a prospective and complementary tool in early patients’ risk stratification, Moura et al. contribution focused on identifying molecular signatures for myelodysplastic syndrome (MDS) progression – a heterogenous group of clonal hematopoietic disorders fundamentally associated with anomalous stem cell differentiation and increased risk of acute myeloid leukemia transformation. Their two-phase study first described a label-free LC-MS/MS quantitative proteomic analysis of bone marrow plasma from 28 patients, 13 diagnosed with MDS with ring sideroblasts (MDS-RS) and 15 with MDS with excess blasts (MDS-EB), and secondly a follow-up with gene expression analysis in bone marrow mononuclear cells from 45 patients with MDS subtypes using qPCR. Together, the results revealed Talin-1 (TLN1) and centrosomal protein of 55 kDa (CEP55) as candidate biomarkers for low- and high-risk MDSs with normal *vs.* abnormal karyotypes, demonstrating how valuable MS-based proteomics can be for prognostic prediction and effective therapeutic strategies in precision medicine.

Indeed, proteome characterization directly from patient samples based on MS has become a ‘must’ in translational research. However, the complexity of proteomics sample preparation protocols still represents a barrier to the technique’s implementation in non-specialist clinical settings. Regarding investigations on closely-related cells, also important for the diagnosis of hemato-oncological disorders such as MDS, van der Pan et al. outlined a three-phase comparative study to refine the ideal sample preparation procedure for MS-oriented proteomic characterization of paucicellular samples. At first, five cell lysis protocols combined with two clean-up techniques were tested on 2,500 to 50,000 cells samples according to their maximum proteome coverage by TMT-based protein quantification and parameters such as reproducibility, ease of use, processing time and cost. Once the best strategy was defined, phase two evaluated its performance on maturational relationships of cell subsets and phase three validated the MS approach sensitivity by high-end spectral flow cytometry. The feasibility of the selected method was verified in 38 different cell types, demonstrating that the original high standards of MS can be diluted in an affordable and user-friendly procedure for clinical and translational laboratories.

Undoubtably, biomarker discovery represents the most widespread translational application of MS nowadays. The power of basic proteomics to discriminate cell lines and improve the prognosis and monitoring of oncological patients was shown by Militaru et al. Their label-free LC-MS/MS-based comparative study evaluated the proteome of 5 metastatic melanoma cell lines, ranging from non-pigmented to highly pigmented, to address the relationship between pigmentation status and melanoma aggressiveness. Correlating the regulation of proteins specifically associated with migration and invasion with cell migration capacity in assays revealed a set of novel biomarker candidates to distinguish aggressive amelanotic tumor cells from less invasive melanotic types. Additionally, the influence of hypoxia on melanoma cells proteome was also verified to simulate microenvironmental conditions during cancer progression and thus consider possible changes induced in gene expression.

MS can also be combined with non-invasive liquid biopsies to ensure a robust yet easy to implement diagnostic tool. Reporting a case-control study of venous thromboembolism (VTE) in non-small cell lung cancer (NSCLC), a common and deadly complication highly incident in these patients, Liu et al. applied data-independent acquisition (DIA) to screen in plasma potential biomarkers and pathways for VTE diagnosis, risk stratification and treatment. Samples from 20 VTE *vs.* 15 non-VTE NSCLC patients were analyzed by combining data-dependent acquisition (DDA) library generation and DIA assay cycles, a proteomics strategy of greater sensitivity, specificity and reproducibility. Five differentially regulated proteins showed promising area under the curve (AUC).

As a versatile tool that transitions from behind the scenes to clinical practice, mass spectrometry-based lipidomics and metabolomics analyzes, also have its highlight in Chalova et al. review about the potential role of short-chain fatty acids (SCFAs) as biomarkers for various diseases, including different types of cancer, as well on how to determine SCFAs by GC, LC or CE-MS. SCFAs are the main metabolites produced by bacterial fermentation of non-digestible carbohydrates in the gastrointestinal tract (intestinal microbiota), representing the leading flow of carbon from the diet. Recent advances in SCFAs sample pretreatment, separation techniques, and detection methods have unlocked the possibility of developing future diagnostic tools based on targeted metabolite profiling.

Collectively, the studies covered by this Research Topic represent a small sample of MS potential as a cutting-edge analytical technique. Its competence in providing instant results and faster feedback has inspired new applications to answer clinical questions and provide insights especially into pathogenesis and treatment, contradicting the idea that MS is too demanding for clinical settings.

## Author contributions

RD: Writing – original draft, Writing – review & editing. AM: Conceptualization, Writing – original draft, Writing – review & editing.

